# Supplementary material for: A comprehensive benchmark of single-cell Hi-C embedding tools
Source: Nat Commun. 2025 Oct 14;16:9119. doi: 10.1038/s41467-025-64186-4 (PMC12521359; doi:10.1038/s41467-025-64186-4)
Supplement: Supplementary file 1 — Supplementary Information [file 41467_2025_64186_MOESM1_ESM.pdf]

A comprehensive benchmark of single-cell Hi-C embedding tools

Dylan Plummer[1,2], Xiuyuan Lang[1,3], Shanshan Zhang[1,3], Yan Li[1], Jing Li[2,4]*, Fulai Jin[1,2,4,5]*

* Corresponding authors: Jing Li (jingli@case.edu); Fulai Jin (fxj45@case.edu).

Supplementary Information (15 supplementary figures, 2 supplementary tables)

Supplementary Figure 1. Overview of the scHi-C preprocessing and embedding options.

Supplementary Figure 2. Average ARI across Resolutions (1Mb-200kb).

Supplementary Figure 3. Full benchmark visualization using UMAP or t-SNE.

Supplementary Figure 4. Additional examples showing that long-range interactions distinguish embryogenesis datasets.

Supplementary Figure 5. Large-scale genome reorganization during embryogenesis from oocyte to 64C embryos.

Supplementary Figure 6. Structural variation at different distances during cell cycle.

Supplementary Figure 7. Random-walk enhances higher order structures.

Supplementary Figure 8. Additional examples showing the impacts of random-walk on embedding tools.

Supplementary Figure 9. Inverse Document Frequency Transformation negatively impact the embedding of neuron subtypes.

Supplementary Figure 10. High resolution improves the Va3DE embedding of neuron subtypes in the human brain atlas data.

Supplementary Figure 11. Runtime vs. Resolution and Cell Count

Supplementary Figure 12. *Va3DE* is a CNN-based variational autoencoder with *Gaussian* mixture prior.

Supplementary Figure 13. The Gaussian mixture prior of Va3DE is best over-parameterized

Supplementary Figure 14. No single clustering algorithm is optimal in all scenarios

Supplementary Figure 15. Low embeddings performance of HiRES and scCARE-seq datasets are not due to low cell counts.

Supplementary Table 1. Benchmark datasets.

Supplementary Table 2. Embedding methods.

Supplementary Figure 1

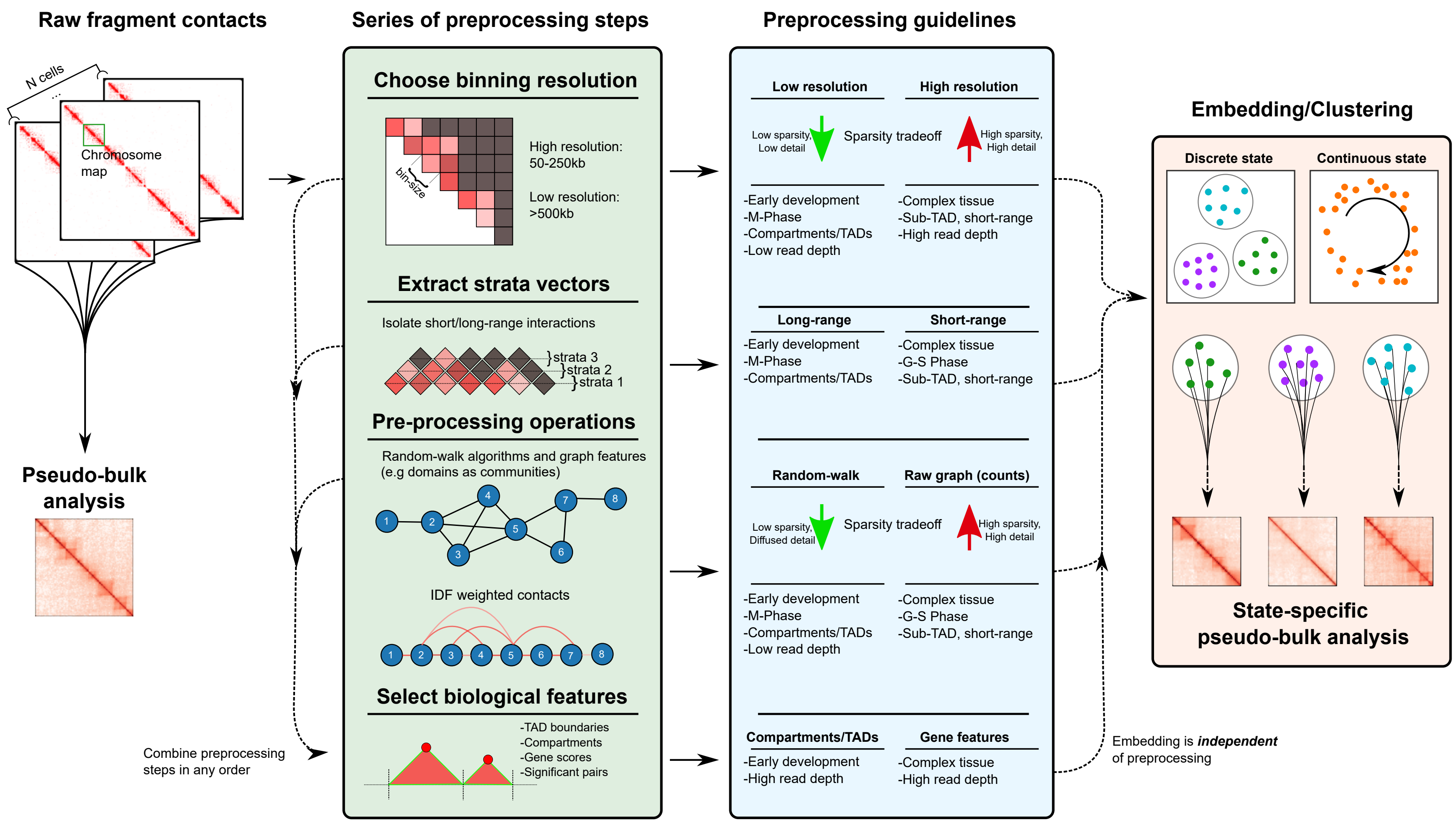

Supplementary Figure 1. Overview of the scHi-C preprocessing and embedding options.

Starting from raw scHi-C contact matrices, we decouple multiple data preprocessing options from the benchmarked tools to represent data at different resolution, to choose min/max interactions distance for consideration, to perform random-walk imputation, and to select biological features such as compartments and TADs. We offer some general guidelines for each option and how it relates to the sparsity tradeoff at the center of scHi-C analysis. After representing data in a feasible way for an embedding method, we can compute low dimensional embeddings and run various clustering algorithms to identify genome architecture heterogeneity across cell states.

Supplementary Figure 2

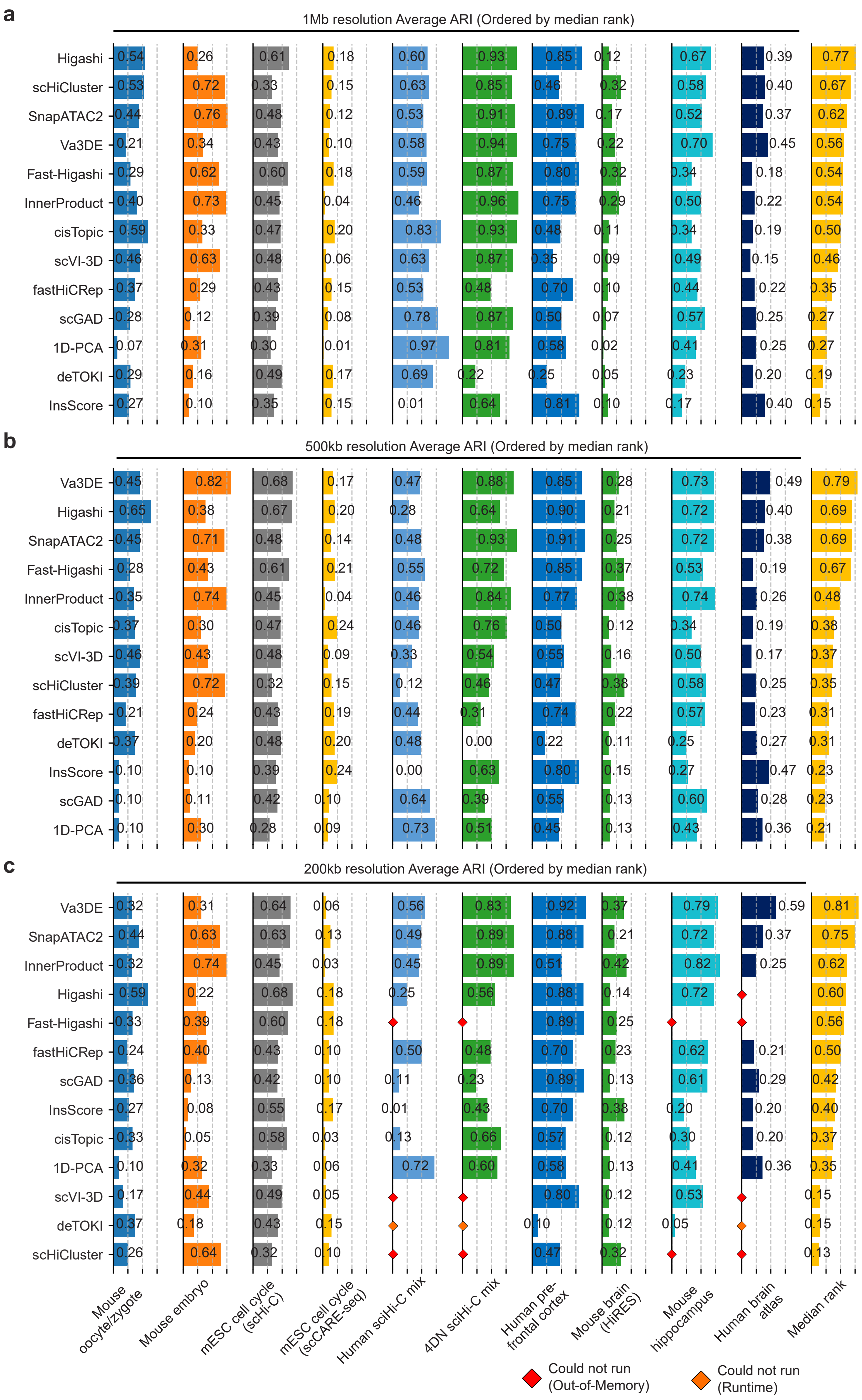

Supplementary Figure 2. Average ARI across Resolutions (1Mb-200kb).

a. Average ARI for each method at 1Mb resolution, ordered by median rank across all datasets. Top performers are *Higashi*, *SnapATAC2*, and *scHiCluster*. **b.** Same ARI analysis at 500kb resolution. Top performers are *Va3DE*, *Higashi*, and *SnapATAC2*. **c.** Same ARI analysis at 200kb resolution. Methods which ran out of memory are indicated by red marks and methods which ran for more than a day before being killed are indicated by orange marks. Both failures are considered an ARI of 0. Top performers are *Va3DE*, *SnapATAC2*, and *InnerProduct*. Source data are provided as a Source Data file.

Supplementary Figure 3

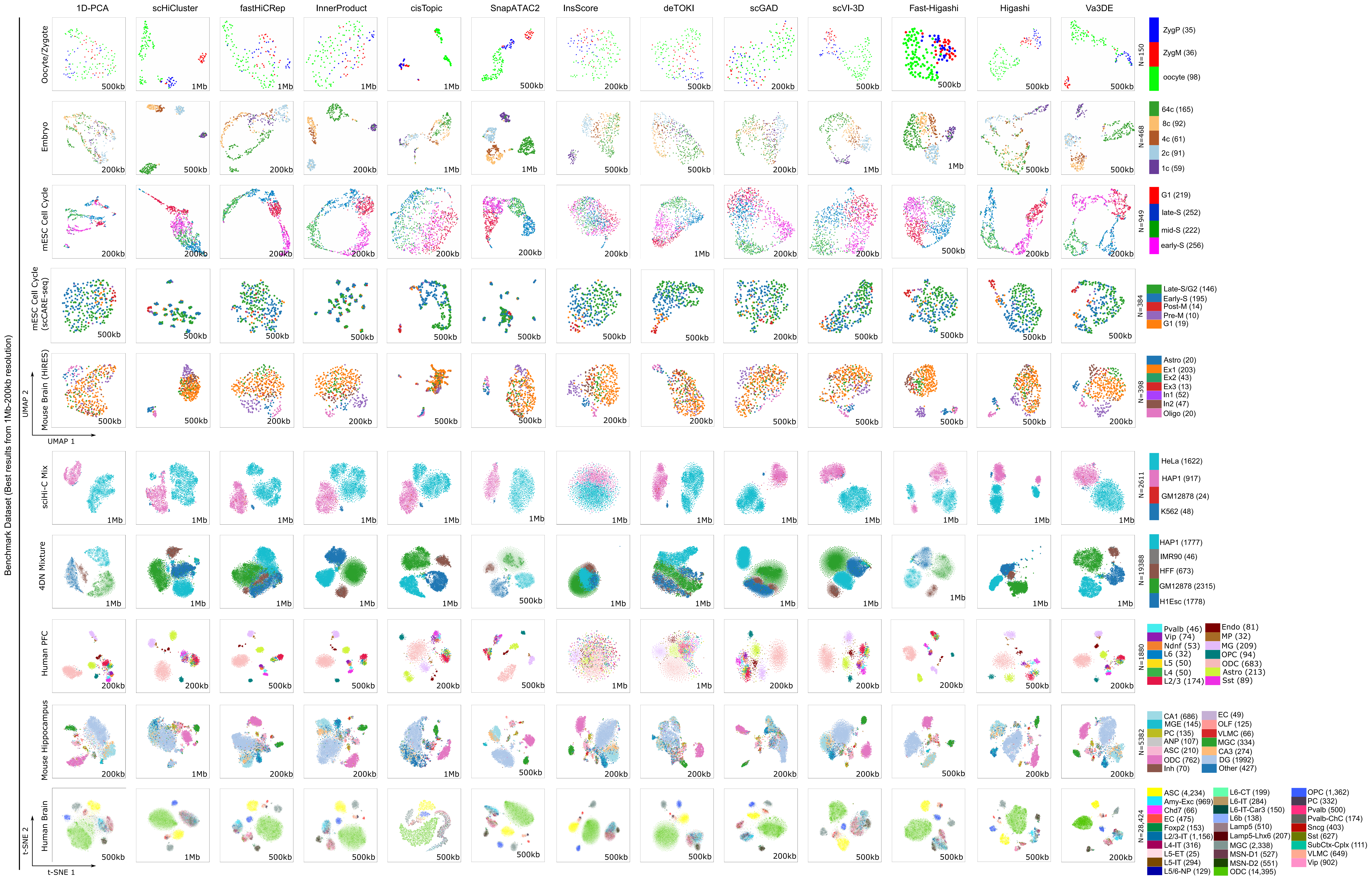

Supplementary Figure 3. Full benchmark visualization using UMAP or t-SNE.

Embedding visualizations of the best results of every method (using default settings) on each dataset included in our benchmark. The resolution used to achieve the best result is displayed in each embedding plot. We use UMAP for the embryogenesis and mESC cell cycle data and use t-SNE for the others based on how well they allowed us to qualitatively confirm the ARI and AvgBIO results in **Figure 1**.

Supplementary Figure 4

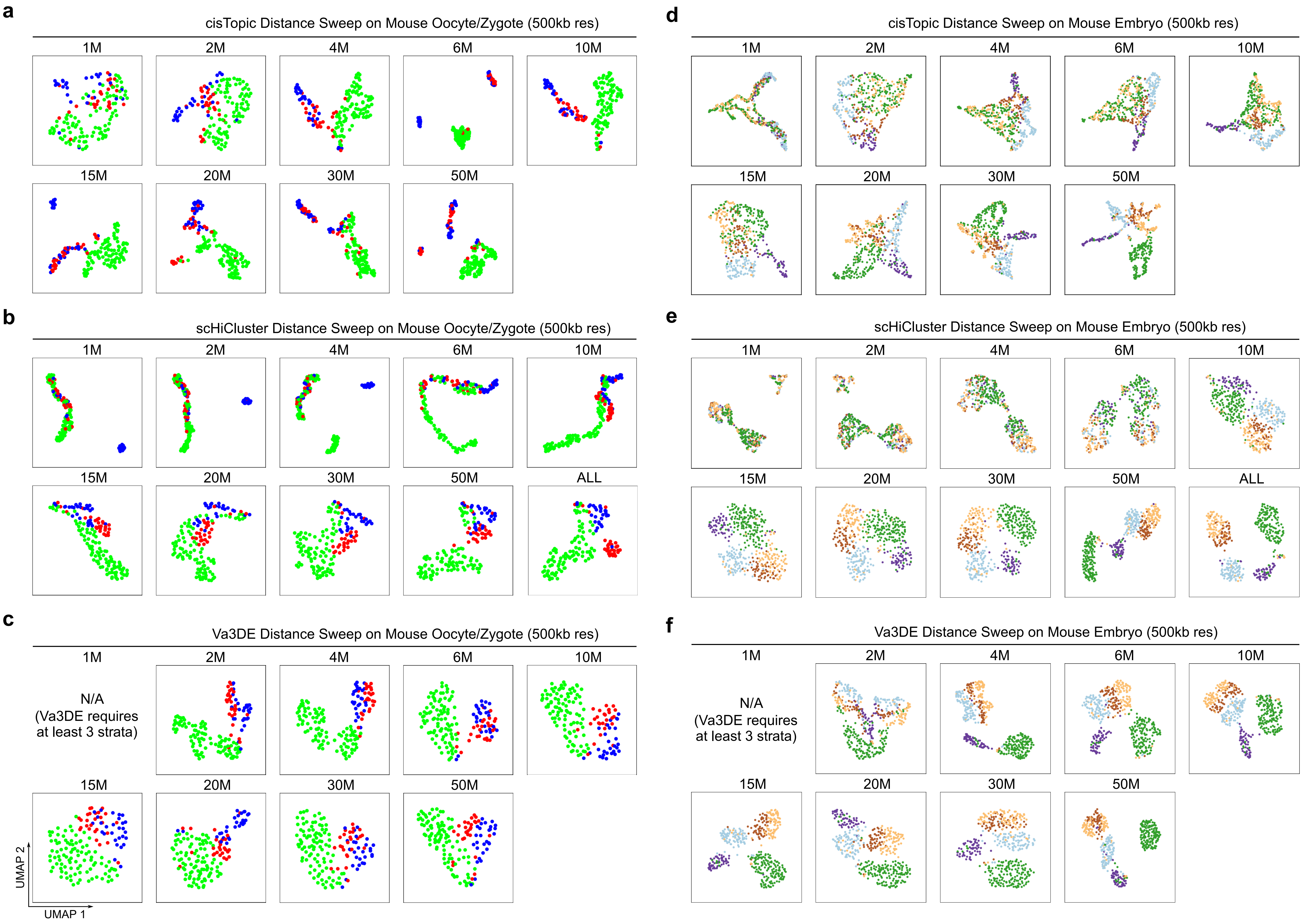

Supplementary Figure 4. Additional examples showing that long-range interactions distinguish embryogenesis datasets.

Related to **Figure 2, a-c.** Embedding mouse oocytes/zygotes at varying maximum distances (from <1Mb to <50Mb) using *cisTopic*, *scHiCluster*, and *Va3DE*. d**-f.** Embedding mouse early embryo cells at varying maximum distances (from <1Mb to <50Mb) using *cisTopic*, *scHiCluster*, and *Va3DE*.

Supplementary Figure 5

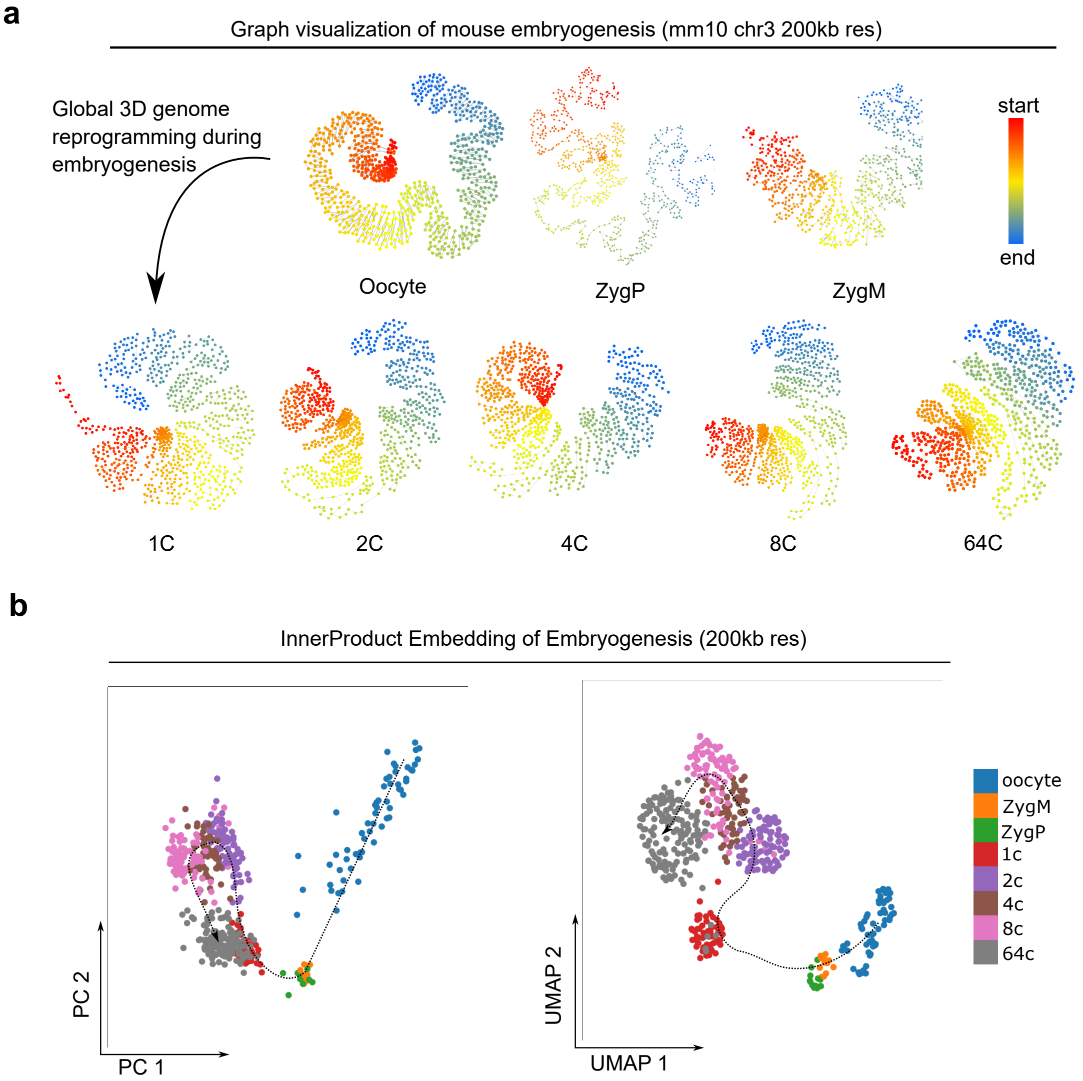

Supplementary Figure 5. Large-scale genome reorganization during embryogenesis from oocyte to 64C embryos.

a. Force-directed graph visualization of mouse embryogenesis pseudo-bulk data from oocyte to 64C embryos. **b.** PCA and UMAP visualization when mouse oocyte/zygote and mouse early embryo datasets are embedded together with *InnerProduct*.

Supplementary Figure 6

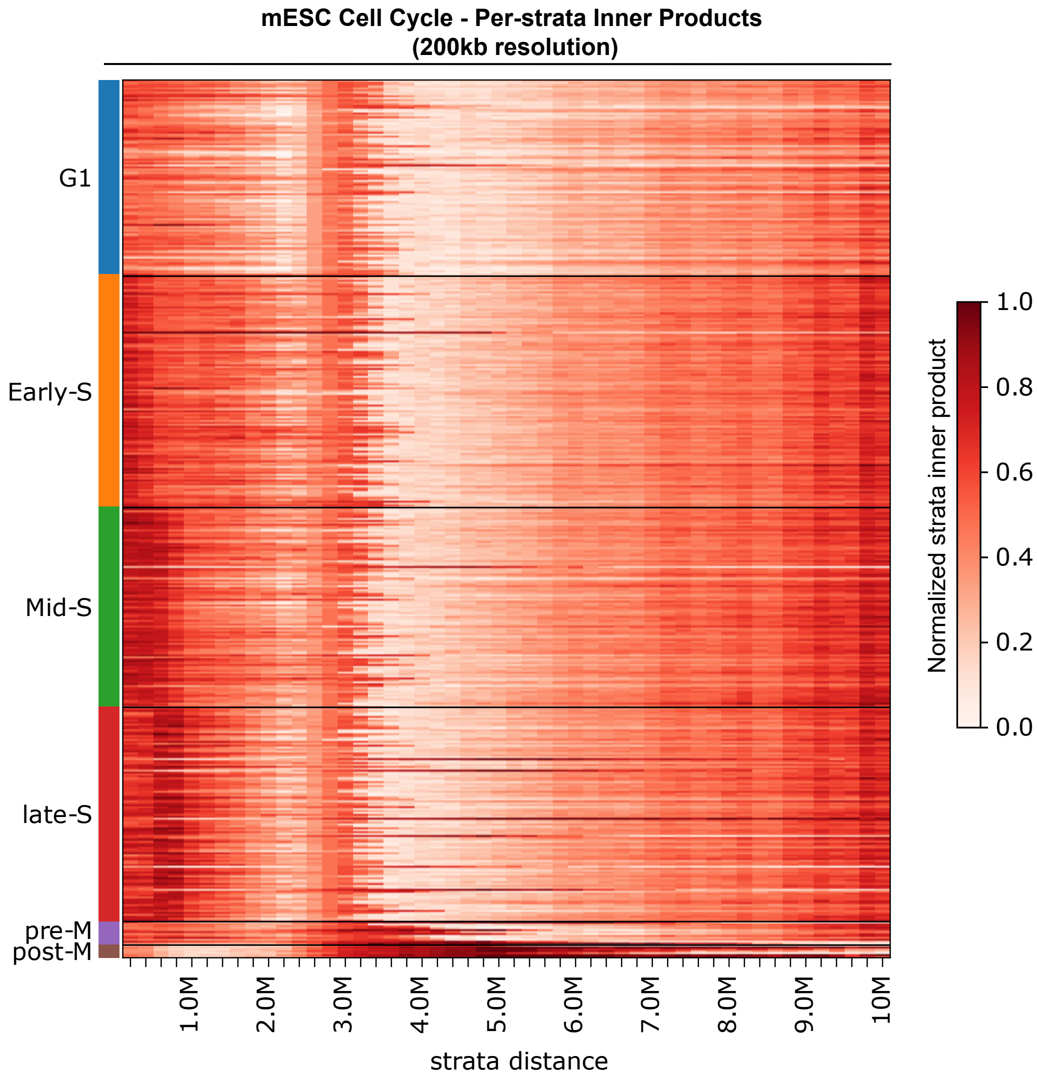

Supplementary Figure 6. Structural variation at different distances during cell cycle.

Per-strata InnerProduct heatmap for mESC cell cycle data analyzed at 200kb resolution from 0-10Mb. Note G1-S phases show obvious variation from 0-1Mb, and M-phase show long-range variation from 2-10Mb.

Supplementary Figure 7

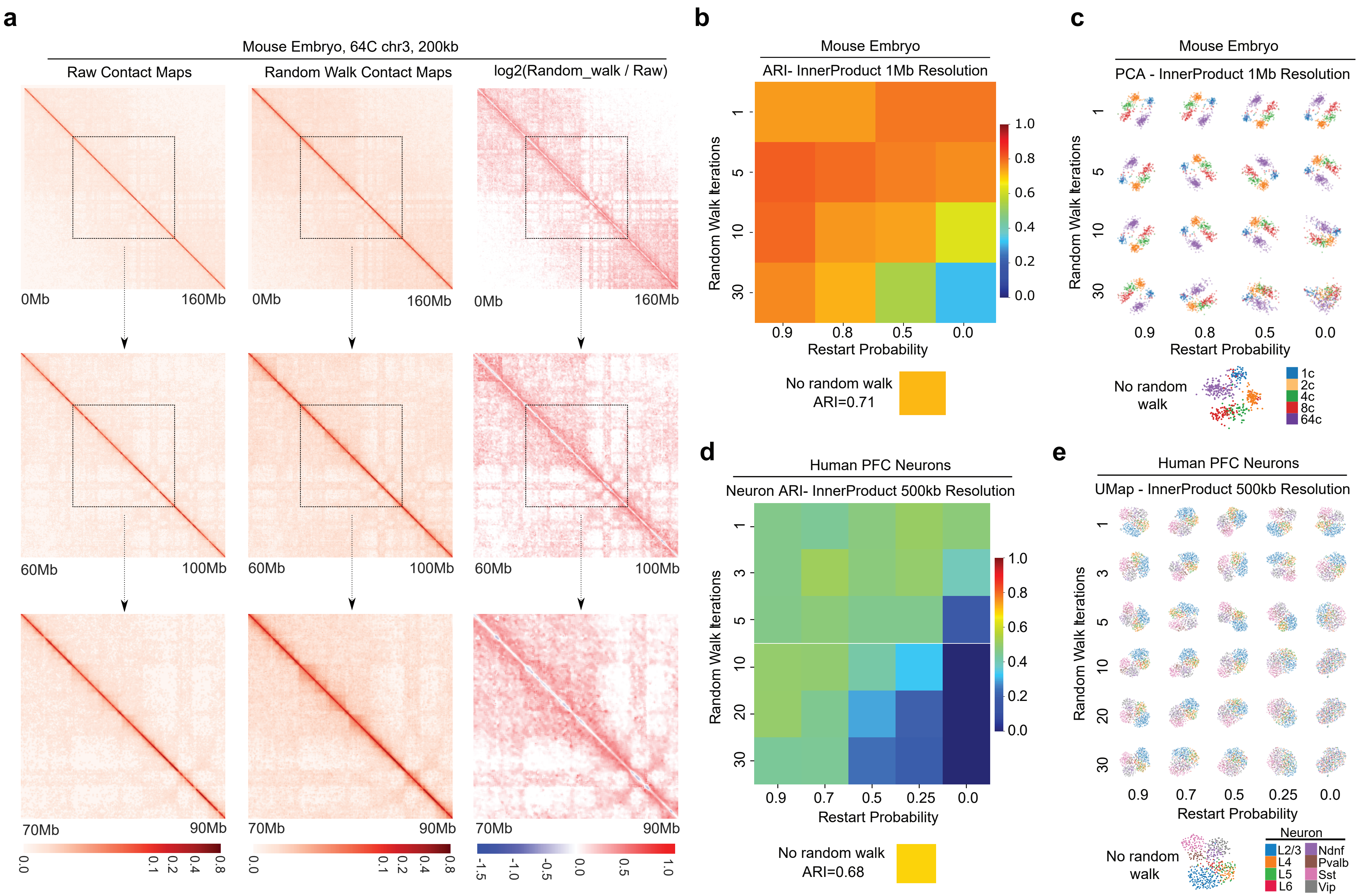

Supplementary Figure 7. Random-walk enhances higher order structures.

a. Left: raw contact maps as contact probabilities. Middle: contact heatmaps after applying random walk imputation process. Right: the ratio between the middle and left heatmaps. Heatmaps from up to bottom continuously zoom into smaller region. We see strengthened signal at both compartment and TAD levels but not near the diagonal. **b**. Assessing how random walk parameters affect the embedding of mouse embryo dataset. Embedding is performed with *InnerProduct* at 1Mb resolution, and we do random walk with different random walk iterations and restart probabilities. ARI results are summarized with heatmap. The color box below shows the ARI when no random walk is performed. Too much random-walk will eventually decrease the ARI. **c**. PCA visualizations of mouse embryo embeddings. Note that too much random walk settings will mix cells from different embryo stages together. **d-e**. Same analysis as in (**a-b**) but with the human PFC data and only measuring the ARI for neuron populations, thus focusing on short-range 3D genome heterogeneity. Note that random walk hurts the neuron cell embedding regardless of the settings. Source data are provided as a Source Data file.

Supplementary Figure 8

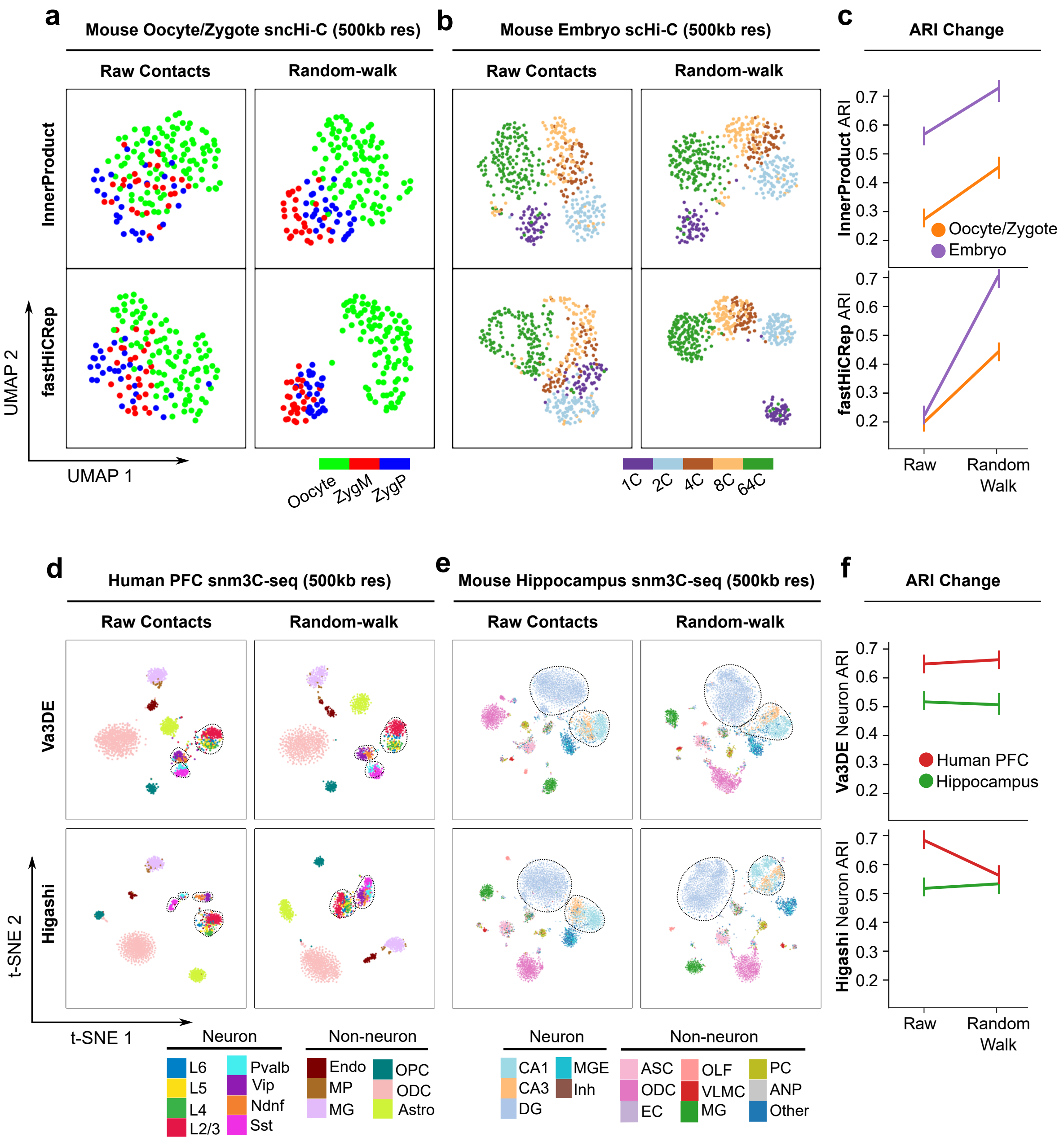

Supplementary Figure 8. Additional examples showing the impacts of random-walk on embedding tools.

a-b. Mouse oocyte/zygote (**a**) and early embryo data (**b**) embedded using *InnerProduct* and *fastHiCRep* with and without random walk preprocessing. **c.** Quantitation of (**a-b**) showing improved ARI after random-walk. **d-e.** Human PFC and mouse hippocampus datasets embedded with deep learning methods *Va3DE* and *Higashi* with or without additional random walk process. Dashed lines circled out neuron subpopulations. **f.** Summary of (**d-e**) showing largely unchanged Neuron ARI except a slight decrease of performance for *Higashi* on human PFC data.

Supplementary Figure 9

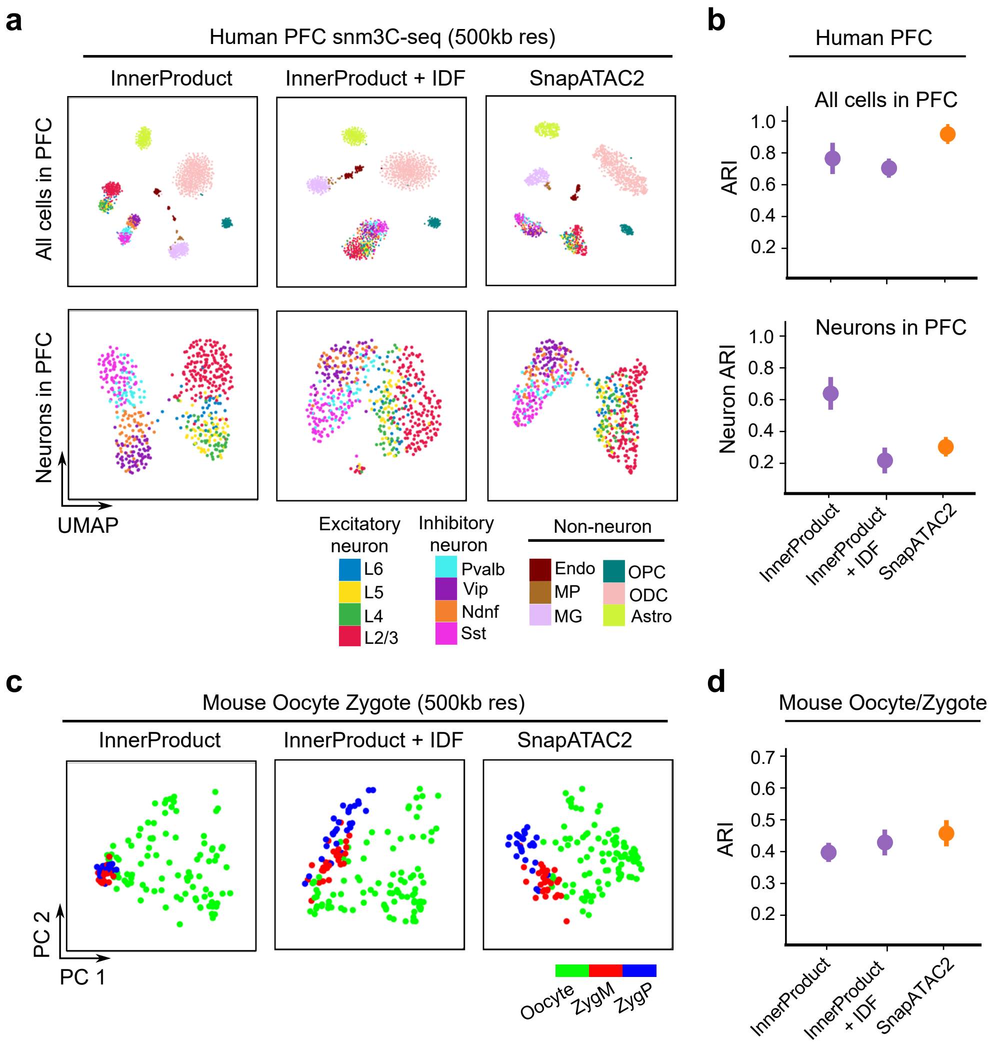

Supplementary Figure 9. Inverse Document Frequency Transformation negatively impact the embedding of neuron subtypes.

a. Human PFC data embedded using *InnerProduct* and *SnapATAC2.* First row: embedding all cell populations in human PFC; second row: visualize the neurons only. Left column: UMAPs of *InnerProduct* embedding using raw contacts; middle column: InnerProduct embedding after IDF transformation; right column: *SnapATAC2* embedding. **b**. Quantify the results from (**a**) quality with ARI for cell types and neuron AIR for neurons only. Note the significantly lower neuron ARI with SnapATAC2 or after adding IDF transformation to InnerProduct. **c-d**. Same analysis as in (**a-b**) but using the mouse oocyte/zygote dataset. Here applying the IDF transformation gives us similar principal components as *SnapATAC2*.

Supplementary Figure 10

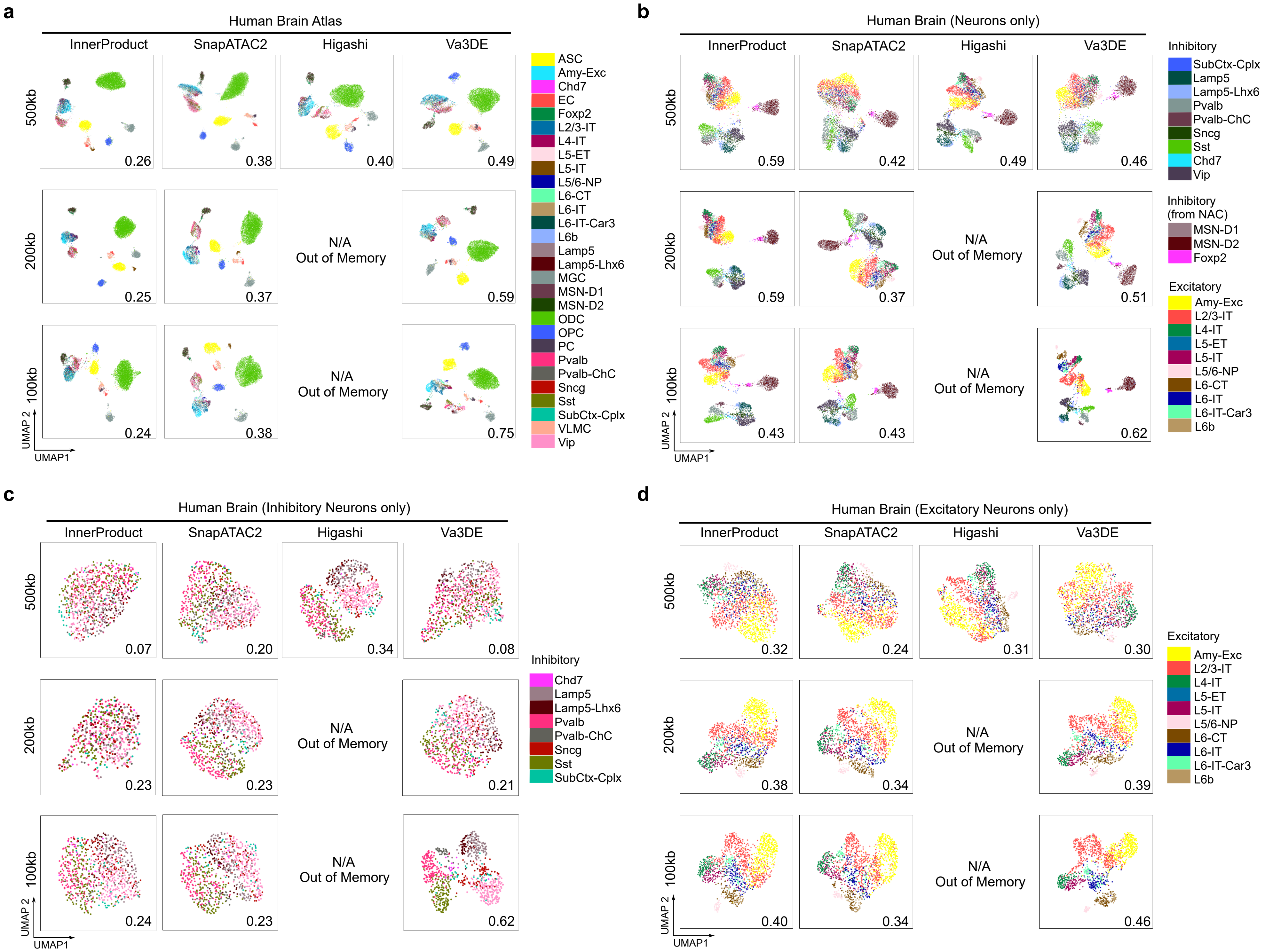

Supplementary Figure 10. High resolution improves the Va3DE embedding of neuron subtypes in the human brain atlas data.

Related to **Figure 6d-g**. UMAPs showing the embedding of human brain atlas data with *InnerProduct, SnapATAC2, Higashi,* and *Va3DE* at 500kb, 200kb, and 100kb resolutions. **a.** all cell types; **b.** all neuron populations; **c**. the inhibitory neurons only; **d**. the excitatory neurons only. The numbers in the UMAPs are ARI values. Note that *Va3DE* shows best embedding of neuron subtypes at 100kb resolution.

Supplementary Figure 11

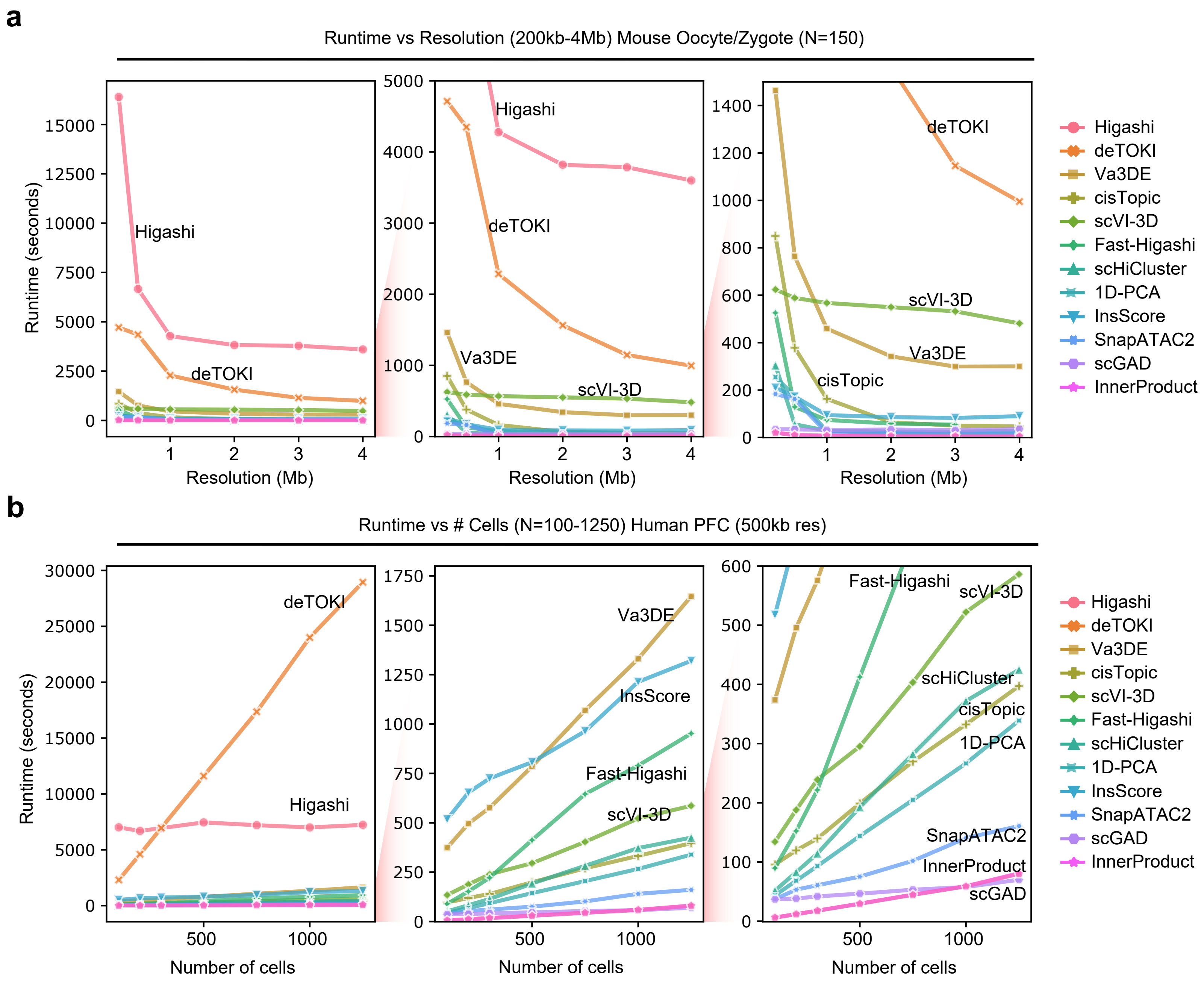

Supplementary Figure 11. Runtime vs. Resolution and Cell Count

a. Runtime of each method from 100kb to 4Mb on the mouse oocyte/zygote dataset. Left shows all methods, middle zooms into only runtimes ~1 hour, and right zooms into only runtimes ~30 minutes. Most methods runtime scales quadratically with resolution. **b.** Runtime of each method on a subsample of the human PFC dataset from 100 to 1250 cells. Left shows all methods, middle zooms into only runtimes ~30 minutes, right zooms into only runtimes ~10 minutes. Most methods runtime scales linearly with the number of cells except *Higashi*. Source data are provided as a Source Data file.

Supplementary Figure 12

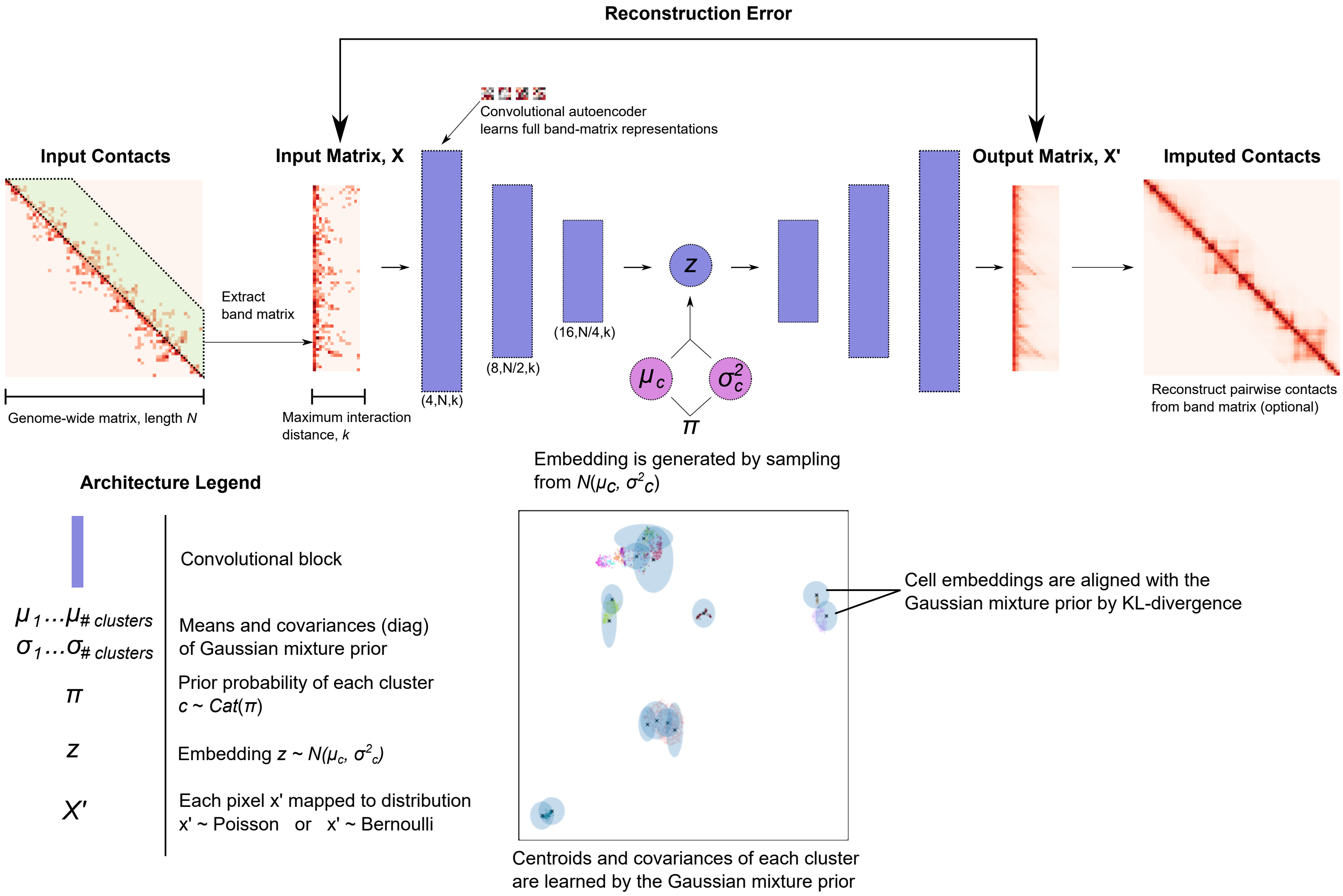

Supplementary Figure 12. *Va3DE* is a CNN-based variational autoencoder with *Gaussian* mixture prior.

Va3DE architecture illustrated with a single input-out contact matrix pair. Starting from the *N* x N genome-wide cis contact matrix, we extract a fixed number of strata and concatenate them into a *k* x *N* band matrix. This band matrix is embedded using a series of convolution and pooling layers, though we only pool over the genome coordinate dimension (vertically), not the strata dimension (horizontally). The output matrix is then decoded using a series of transpose convolution layers. The final prediction is sampled from either a *Poisson* distribution if the input is raw counts, or a *Bernoulli* distribution if any other input is provided. The embedding for each matrix is sampling from a *Gaussian* mixture model prior to enforce the cell type population clustering structure we wish to take advantage of. The centroid categorical probabilities, locations, and covariances, are all learnable parameters of the model. We train *Va3DE* to optimize the ELBO which combines the reconstruction error between the input band matrix and the output sampled contacts with the KL-divergence between the cell embeddings and the learned Gaussian mixture prior.

Supplementary Figure 13

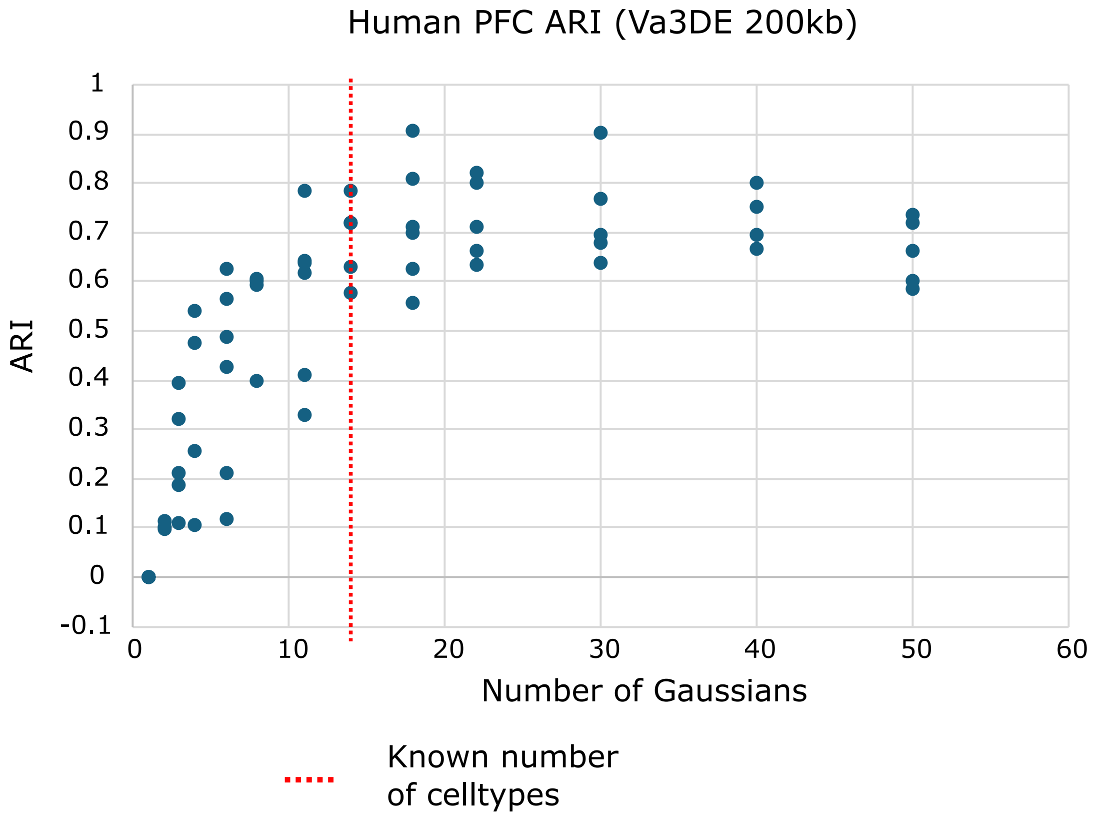

Supplementary Figure 13. The Gaussian mixture prior of Va3DE is best over-parameterized

We train *Va3DE* on the human PFC dataset at 200kb resolution varying only the number of gaussians. The dataset has 14 known cell types. We observe an increase in performance as the number of gaussians approaches this true number of clusters, but we find that the performance remains constant beyond 14 gaussians. This suggests that it is better to over-estimate than under-estimate the number of cell types when applying Va3DE to a new scHi-C data. Source data are provided as a Source Data file.

Supplementary Figure 14

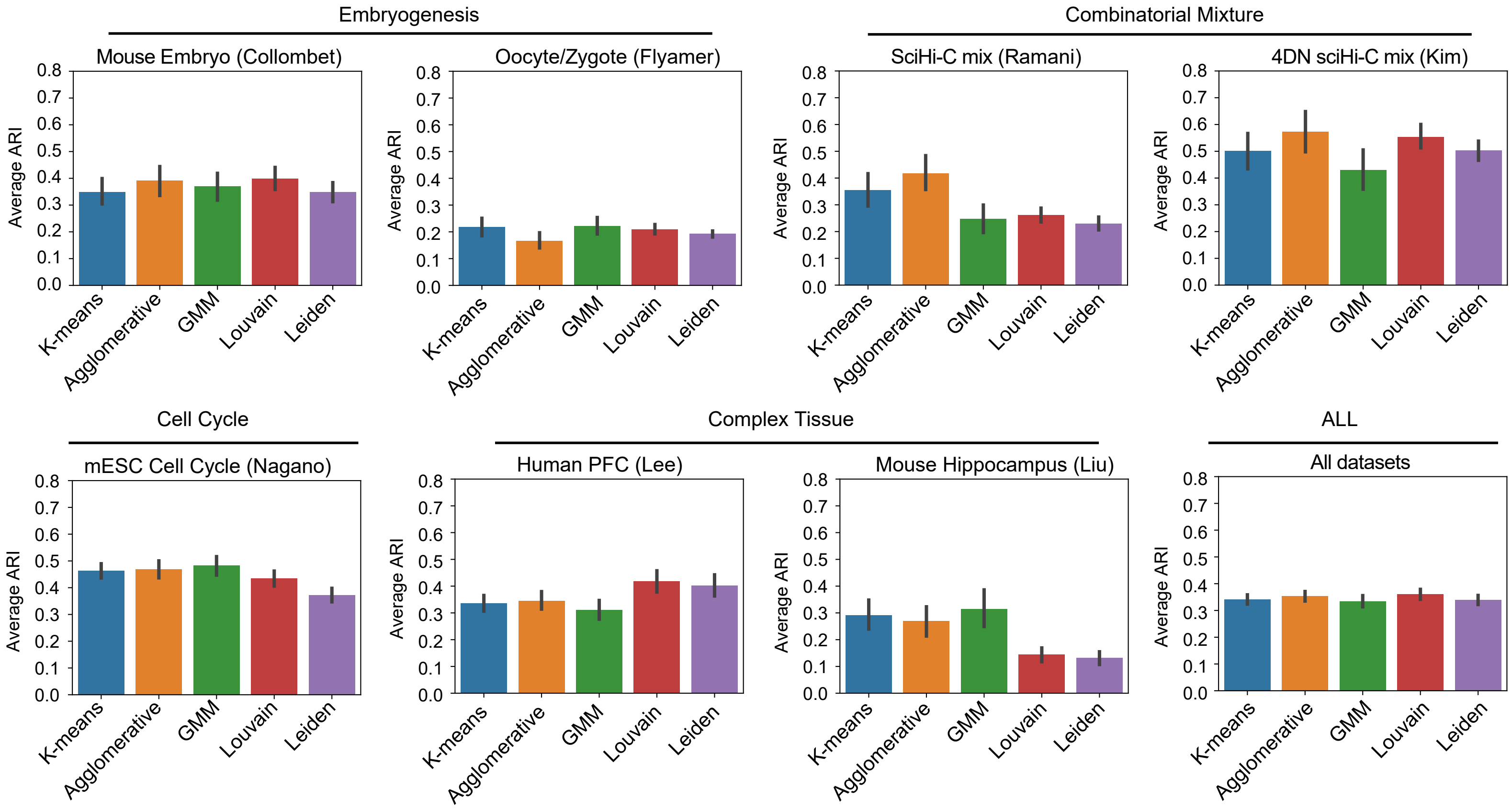

Supplementary Figure 14. No single clustering algorithm is optimal in all scenarios

Bar plots show the average ARI across all methods in each dataset using the five clustering algorithms. Note that some clustering algorithms (such as Leiden and Louvain) have variable performance across different datasets. Overall, none of the clustering methods is a clear winner across all datasets. Source data are provided as a Source Data file.

Supplementary Figure 15

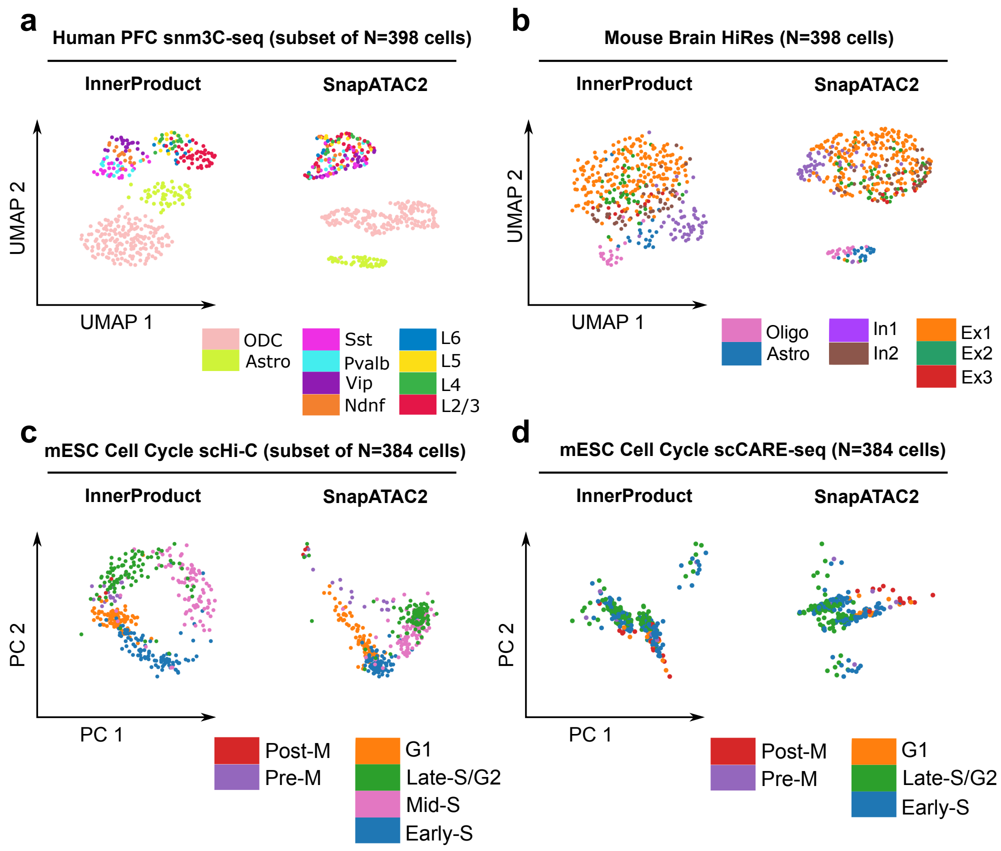

Supplementary Figure 15. Low embeddings performance of HiRES and scCARE-seq datasets are not due to low cell counts.

a. Embeddings of a random subset (n=398 cells) of the human PFC snm3C-seq dataset using *InnerProduct* and *SnapATAC2*; **b**. Embeddings of the mouse brain HiRES dataset (n=398) using *InnerProduct* and *SnapATAC2.* When the cell number is the same, the human PFC dataset is still easier to cluster; **c.** Embeddings of a random subset (n=398 cells) of the mESC cell cycle scHi-C dataset using *InnerProduct* and *SnapATAC2*; **d.** Embedding the mESC cell cycle scCARE-seq dataset (n=398). The embedding of scCARE-seq cell cycle data does not yield meaningful clusters.

Supplementary Table 1. Benchmark datasets.
Ten scHi-C datasets included in this study indicating the number of cells, number of celltypes, average cis contacts, source of celltype annotations, default maximum interaction distance used, recommended resolution for analysis, the main source of celltype heterogeneity, and recommended preprocessing steps.

Sample	Reference	# Cells	# Cell types	Avg Cis Contacts	Source of Cell ID	Default Max Distance (if truncating)	Recommended Resolution	Main source of heterogeneity	Recommended Preprocessing
Mouse Oocyte/Zygote	Flyamer et al (snHi-C)	169	3	141,677	pre-determined	<10Mb	500kb-1Mb	Long-range interactions (>5Mb)	Random-Walk
Mouse Embryo	Collombet et al (scHi-C)	468	5	193,094	pre-determined	<10Mb	500kb-1Mb	Long-range interactions (>5Mb)	Random-Walk
Mouse Cell Cycle	Nagano et al (scHi-C)	1,560	6	119,236	in silico phasing	<10Mb	100-500kb	Fraction of short range vs long range	None
Mouse Cell Cycle	Qu et al (scCARE-seq)	384	4	96,010	in silico phasing	<10Mb	100-500kb	Fraction of short range vs long range	None
Human sciHi-C Mixture (GM12878, HAP1, HeLa, K562)	Ramani et al (sciHi-C)	2,611	4	19,695	pre-determined	<10Mb	500kb-1Mb	Varies at all scales	None
Human 4DN sciHi-C Mixture: (GM12878, H1Esc, HFF, IMR90, HAP1)	Kim et al (sciHi-C)	19,388	5	5,987	pre-determined	<10Mb	500kb-1Mb	Varies at all scales	None
Human Prefrontal Cortex (PFC)	Lee et al (snm3C-seq)	4,236	14	78,887	co-assay m3C clustering	<2Mb	100-500kb	Short-range interactions (<2Mb)	Distance truncation (<2Mb)
Mouse Brain	Liu et al (HiRES)	399	7	522,807	co-assay scRNA clustering	<2Mb	100-500kb	Short-range interactions (<2Mb)	Distance truncation (<2Mb)
Mouse Hippocampus	Liu et al (snm3C-seq)	5,382	13	150,014	co-assay m3C clustering	<2Mb	100-500kb	Short-range interactions (<2Mb)	Distance truncation (<2Mb)
Human Brain Atlas	Tian et al (snm3C-seq)	32,177	29	183,103	co-assay m3C clustering	<2Mb	100-500kb	Short-range interactions (<2Mb)	Distance truncation (<2Mb)

Supplementary Table 2. Embedding methods.

13 embedding methods tested in this study including the scHi-C data representation used, the base dimension reduction algorithm, the default preprocessing (if applicable), the memory compleixty in terms of number of cells, resolution, and interaction distance considered, the main memory bottleneck of each method, whether the method can utilize GPU acceleration, whether the method must load all chromosomes into memory at once, whether the method utilizes sparse matrix data structures, whether it can process contact maps in parallel, the resolutions we were able to benchmark, the recommended resolution, any additional preprocessing that we tested, the interaction distance ranges that we tested, the recommended interaction distance to use, the default number of strata used, the batch size (for deep learning), the learning rate (for deep learning), additional hyperparameters, and the main datasets that were tested by each method in its original publication.

Method	Data Representation	Dimension Reduction	Default Preprocessing	Memory Complexity	Main Memory Bottleneck	Use GPU	Load the contacts of All Cells in RAM	Load all Chroms in RAM	Sparse Representation	Contact Maps Processed in Parallel	Resolution tested	Recommended Resolution	Additional preprocessing tested in this study	Distances tested	Recommended Distance	Default # of strata	Batch size	Learning rate	Other hyperparameters	Datasets tested in the original paper
1D-PCA	Raw Cis Visibility	PCA	None	O(min(N, M) * M)	PCA decomposition	☒	☑ Single strata	☒	☒ Dense visibility vectors	☑	50kb-1Mb	100-500kb	VC_SQRT_norm, convolution, random walk	n/a	n/a	32	ALL	n/a	None	n/a
scHiCluster	Full Cis Matrix	PCA	VCSQRT norm + Convolution + Random Walk	O(min(N, M^2) * M^2)	PCA decomposition	☒	☑ Full matrix	☒	☒ Dense matrices	☑	200kb-1Mb	500kb-1Mb	Raw, distance truncation	<200kb - ALL	ALL	ALL	ALL	n/a	None	Mouse Oocyte/Zygote, Human sciHi-C Mixture
FastHiCRep	Stratified (distally weighted)	MDS	Distance truncation	O(N^2 + Mk)	Similarity matrix calculation	☒	☑ Multiple strata	☒	☒ Dense strata vectors	☑	50kb-1Mb	100-500kb	VC_SQRT_norm, convolution, random walk	<50kb - ALL	<10Mb	32	ALL	n/a	None	mESC Cell Cycle;Mouse Oocyte/Zygote
InnerProduct	Stratified (uniformly weighted)	MDS	Distance truncation	O(N^2 + Mk)	Similarity matrix calculation	☒	☑ Multiple strata	☒	☒ Dense strata vectors	☑	50kb-1Mb	100-500kb	VC_SQRT_norm, convolution, random walk, IDF	<50kb - ALL	<10Mb	32	ALL	n/a	None	mESC Cell Cycle, Mouse Oocyte/Zygote, Human sciHi-C Mixture
cisTopic	Locus-pair set	LDA	Distance truncation	O(NMk)	Constructing locus-pair set	☒	☑ Multiple strata	☑	☑ Sparse interactions after preprocessing	☑	50kb-1Mb	100-500kb	VC_SQRT_norm	<50kb - <50Mb	<10Mb	32	ALL	n/a	None	Human 4DN sciHi-C Mixture, Mouse Oocyte/Zygote, mESC Cell Cycle
SnapATAC2	Locus-pair set	Spectral Embedding	TF-IDF	O(NMk)	Constructing locus-pair set	☒	☑ Multiple strata	☑	☑ Sparse interactions after preprocessing	☑	50kb-1Mb	100-500kb	None	<50kb - <50Mb	<10Mb	32	ALL	n/a	Size of variable feature set (default=500k)	Human 4DN sciHi-C Mixture; Human PFC
Fast-Higashi	Hypergraph	PARAFAC	VCSQRT norm + Convolution + Random Walk	O(NM^2)	Constructing hypergraph	☑	☑ Multiple strata	☑	☑ Sparse hyperedges after preprocessing	☒	100kb-1Mb	500kb-1Mb	Raw	<50kb - <50Mb	<10Mb	100	ALL	n/a	L2 and/or read-depth normalization (default=L2 only)	Human sciHi-C Mixture, Human 4DN sciHi-C Mixture, Human PFC, Mouse Hippocampus, Human Brain
scGAD	Gene-body	PCA	BandNorm	O(NM2)	Submatrix of largest gene	☒	☑ Single gene submatrix	☒	☒ Dense gene submatrices	☑	50kb-1Mb	50-500kb	None	n/a	n/a	n/a	ALL	n/a	Read-depth correction	Human PFC;Human sciHi-C Mixture
InsScore	Domain boundaries	PCA	Distance truncation	O(NM + k)	Constructing insulation vectors	☒	☒ One cell at a time	☒	☒ Dense matrices	☒	200kb-1Mb	500kb-1Mb	n/a	n/a	n/a	32	ALL	n/a	None	n/a
deTOKI	TAD-like boundaries	PCA	Distance truncation	O(NM + k)	Constructing boundary vectors	☒	☒ One cell at a time	☒	☒ Dense matrices	☑	200kb-1Mb	500kb-1Mb	n/a	n/a	n/a	32	ALL	n/a	None	n/a
scVI-3D	Stratified (uniformly weighted)	Generative Neural Network	BandNorm + Distance truncation	O(NMk)	Constructing band matrices	☑	☑ Multiple strata	☒	☒ Dense band matrices	☒	50kb-1Mb	500kb-1Mb	n/a	<50kb - <50Mb	<10Mb	32	128	0.001	Read-depth correction (default=False)	Human sciHi-C Mixture, Human 4DN sciHi-C Mixture, Human PFC
Higashi	Hypergraph	Graph Neural Network	None	O(NM^2)	Constructing hypergraph	☑	☑ Full matrix	☑	☑ Sparse hyperedges after preprocessing	☒	50kb-1Mb	500kb-1Mb	VC_SQRT_norm, convolution, random walk	n/a	n/a	ALL	128	0.001	None	Human sciHi-C Mixture, Human 4DN sciHi-C Mixture, Human PFC, mESC Cell Cycle
Va3DE	Full Cis Matrix (truncated)	Generative Neural Network	Distance truncation	O(Mk)	Constructing band matrix	☑	☒ Batches of cells	☑	☒ Dense band matrices	☒	50kb-1Mb	100-500kb	VC_SQRT_norm, convolution, random walk, distance	<50kb - <50Mb	<10Mb	32	64	0.0001	Interaction counts or binary (default=binary)	n/a
